# Annatto-Enriched Egg Improves the Perception of Satiety in Healthy Adults—Randomized Clinical Trial: EGGANT Study

**DOI:** 10.3390/foods13050731

**Published:** 2024-02-28

**Authors:** Yeisson Galvis-Pérez, Keilly Pineda, Juliana Zapata, Juan Aristizabal, Alejandro Estrada, María Luz Fernández, Jacqueline Barona-Acevedo

**Affiliations:** 1 Research Group of Toxinology, Food and Therapeutic Alternatives, Universidad de Antioquia UdeA, Medellín 050010, Colombia; yeisson.galvis@udea.edu.co (Y.G.-P.); keilly.pineda@gmail.com (K.P.); juliana.zapatam@udea.edu.co (J.Z.); 2 School of Microbiology, Universidad de Antioquia UdeA, Medellín 050010, Colombia; 3 Physiology and Biochemistry Research Group-PHYSIS, Universidad de Antioquia UdeA, Medellín 050010, Colombia; juan.aristizabal@udea.edu.co; 4 School of Nutrition and Dietetics, Universidad de Antioquia UdeA, Medellín 050010, Colombia; alejandro.estrada@udea.edu.co; 5 Demography and Health Research Group, Universidad de Antioquia UdeA, Medellín 050010, Colombia; 6 School of Nutritional Sciences and Wellness, The University of Arizona, Tucson, AZ 85721, USA; mlfernandez@arizona.edu

**Keywords:** egg, annatto, ghrelin, satiety, healthy adults

## Abstract

Diet is one of the factors that prevents the development and death from cardiovascular diseases (CVD). It has been proposed that diets high in protein, which increase satiety, and with a high content of antioxidants, help reduce cardiovascular risk factors. The egg is one of the foods that produces greater satiety and provides antioxidants. In addition, due to its lipophilic matrix, it could improve the bioavailability of other dietary antioxidants such as Annatto. Objective. This study evaluated the effects of egg and annatto-enriched egg consumption on satiety markers and CVD risk factors in healthy adults from Colombia. Methods. A parallel randomized clinical trial was conducted, where one hundred and five (*n* = 105) men and women, divided into three groups, consumed daily for 8 weeks: (a) two eggs (egg group), or (b) two eggs with annatto (egg + annatto group), or (c) two egg whites (placebo group). RESULTS. The three groups were similar in gender distribution. No significant changes were found over time (before vs. after) in any of the groups nor between the groups in anthropometric variables, physical activity, eating profile, and ghrelin as an objective marker of satiety. In the egg + annatto group, subjective satiety increased (effect size 0.431; *p* < 0.05) after consumption. Conclusions. In healthy adults, the intake of two eggs, or two eggs with annatto daily for 8 weeks, did not result in significant changes in ghrelin; but eggs with annatto tend to increase the perception of satiety.

## 1. Introduction

The highest percentage of deaths from cardiovascular disease (CVD) occurs in low-middle-income countries and this trend is associated with lifestyles [1]. Therefore, most CVD can be prevented by acting on behavioral risk factors, such as excessive food consumption and physical inactivity [1,2]. The risk of developing CVD grows with the increase in obesity; in Colombia according to the national survey of nutritional situation (ENSIN-from Spanish, Encuesta Nacional de la Situación Nutricional-2015), excess weight in adults (18–64 years) increased by 5.2% from 2010 to 2015 [3].

The consumption of high-protein diets has been proposed as a strategy that can help combat obesity, since proteins have a greater satiety effect than fats and carbohydrates [4]. Protein intake has been found to be associated with lower levels of ghrelin, a hormone that stimulates food intake, which is why it is called the hunger hormone [5].

Egg protein is considered the most bioavailable and complete and is one of the least expensive sources of animal protein. A large egg (50 g) provides about 6 g of high-quality protein, and essential nutrients, such as: all 9 essential amino acids, vitamins (D, A, E, B2, B3, B5, B6, B7, B9, B12), minerals (iodine, zinc, selenium, phosphorous), choline, and the carotenoids: lutein and zeaxanthin; with the advantage of being a low-energy food (only 70 calories), and dense in nutrients [6,7].

Egg protein, especially yolk protein, has a significantly greater satiety effect than other protein sources [8]. Studies show that individuals reduce their caloric intake after an egg breakfast compared to a high-carbohydrate breakfast, lose weight, and experience greater changes in satiety hormones, better controlling their appetite [9]. For example, people who ate breakfast with eggs were less hungry and more satisfied 3 and 24 h after, compared to those who ate breakfast without eggs. Likewise, an increase in ghrelin was observed in the control group compared to the egg breakfast group [9].

Another important component of eggs are carotenoids, which are highly bioavailable given the lipophilic matrix provided by eggs. This could also improve the availability of carotenoids from other foods and thus reduce cardiovascular risk by increasing the absorption of antioxidants from the diet. A low-priced and highly available source of carotenoids that has gained importance in recent years is annatto (*Bixa orellana* L.), which, in addition to being used as a natural colorant, has been attributed healing, anti-inflammatory, antioxidant, and antimicrobial activities [10,11,12]. Annatto supplementation studies conducted in animals such as rabbits, rats, and dogs have reported promising results [13,14,15], and studies in humans have shown its antioxidant potential to reduce oxidative stress [16,17]. However, some review articles suggest that more in vivo studies are needed to evaluate its nutritional and healing properties [18].

As Colombia is a low- to medium-income country [19], the economic capacity of Colombians to acquire a balanced diet, which includes quality protein, abundant fruits and vegetables, and healthy fats, is reduced. As described before, the egg represents a great option to be included in a balanced diet. However, the egg has been controversial due to its cholesterol content. For many years, cholesterol and egg consumption were limited because some studies had associated it with increased blood cholesterol and cardiovascular risk [20]; but given the robust scientific evidence that egg consumption does not increase risk factors for heart disease [21,22], the Dietary Guidelines for Americans do not list cholesterol as a nutrient of concern and have removed dietary cholesterol limits since 2015 [23]. Therefore, this study aimed to evaluate the effects of egg consumption and annatto-enriched egg on satiety in healthy adults from Colombia. The hypothesis of this study was that consumption of eggs and eggs enriched with annatto (*Bixa orellana* L.), compared to egg whites, would improve the subjective perception of satiety, and reduce ghrelin levels in the studied population.

## 2. Materials and Methods

### 2.1. Type of Study

Parallel randomized clinical trial.

### 2.2. Study Population

One hundred five (*n* = 105) men and women, distributed in 3 groups each with *n* = 35, matched by age, sex, and body mass index (BMI). The sample size is based on a mean difference found in the change value of the serum lutein variable (95% confidence, 1:1 ratio, and 85% power) in a similar study [24].

### 2.3. Inclusion Criteria

Age: 18 to 59 years; BMI between 18.5 and 29.9 kg/m^2^.

### 2.4. Exclusion Criteria

Altered values in blood lipids and glucose, or high blood pressure. The use of medications or dietary supplements, pregnant or lactating women, or egg intolerance.

### 2.5. Intervention

The recruitment of participants was carried out between October 2019 and December 2020. After signing the informed consent, and a washout period of 2 weeks, volunteers were randomly assigned to consume one of the following foods daily for 8 weeks: 2 eggs, 2 eggs with annatto (1.2 mg of bixin/kg of body weight), or 2 egg whites (control group). At the beginning and end of the treatment, blood samples were obtained, weight, height and waist circumference were recorded. Additionally, diet intake, satiety and physical activity records were obtained. The number of eggs consumed daily by the participants were based on (a) the current Dietary Guidelines for Colombians (2015) which recommend consuming only one egg a day [3]; therefore, we wanted to challenge and double that amount; and (b) several studies demonstrating positive effects after egg consumption have used an average of two eggs [25,26,27,28].

### 2.6. Procedure for Measuring Height

The subject was asked to place the soles of the feet on the insoles of the stadiometer (SECA 216, Seca S.A.S., Hamburg, Germany). The stadiometer square was slid smoothly and firmly until it touched the crown of the subject’s head [29].

### 2.7. Procedure for Measuring Body Weight with a Portable Scale

The scale (SECA 813, Seca S.A.S., Hamburg, Germany) was turned on and its adjustment to zero (0) was verified. The subject was asked to step on the scale and place the soles of the feet on the scale’s insoles. The subject was asked to remain still during the procedure with the weight of the body distributed on both feet. The data was read on the digital screen of the scale [29].

### 2.8. Body Mass Index (BMI)

BMI was calculated from the weight and height data according to the equation: weight (Kilograms)/height (meters)^2^. With these values, a classification was carried out according to the cut-off points of the WHO [30].

### 2.9. Procedure for Measuring the Abdominal Perimeter

The waist circumference was measured with a tape (Lufkin W606PM, Crescent Tools, Sparks, MD, USA at the upper edge of the iliac crest, applying gentle pressure to the skin, without compressing the tissues and at the end of a normal expiration [29].

### 2.10. Physical Activity

Participants were asked to register the minutes/hours dedicated to performing physical activities, and their intensity, during 7 consecutive days before and during the last week of the intervention. With this information, the metabolic equivalents of task (Mets)/week were calculated according to the compendium of physical activities [31].

### 2.11. Blood Pressure (BP)

Participants were asked to sit for 5 min without talking or moving with feet flat on floor and back supported. An automated upper-arm cuff BP measurement device was used (Omron, Healthcare Inc., Hoffman Estates, IL, USA), with the cuff on the patient’s right upper arm at the level of the heart, following the instructions of the American Heart Association [32]. Two measurements of systolic and diastolic BP were registered separated by 1 min, and the average of the two readings was used for the analysis.

### 2.12. Diet

Diet was analyzed by means of a food frequency questionnaire before starting and in the final week of the study. These records were analyzed to estimate energy (kilocalories) and macronutrient (grams) intake using a Colombian food database developed and validated by nutritionists from the School of Nutrition and Dietetics of the University of Antioquia [33].

### 2.13. Blood Collection

Blood samples were collected from the antecubital vein using dry tubes after an overnight 12 h fast. After 30 min, the tubes were centrifuged at 450× *g* for 15 min to obtain serum, which was aliquoted and frozen at −70 °C for analysis.

### 2.14. Blood Lipids and Glucose

These serum markers were measured at baseline and end of intervention using automated protocols. Detailed information can be found in our previous publication [34].

### 2.15. Ghrelin Measurement

The Human Ghrelin ELISA Kit (Ghrelin-28) (ab263887) was used, following the manufacturer’s recommendations (Abcam, Boston, MA, USA). The test quantifies serum ghrelin by immunoenzymatic assay. The results were calculated using the standard curve of known samples [35].

### 2.16. Visual Analogue Scale (VAS) for Satiety Assessment

Each VAS questionnaire consisted of eight open-ended questions about hunger, satisfaction, fullness, prospective food intake, and taste preference for salty, sweet, savory, or fatty foods. The VAS was a 10 cm line, ranging from “not at all” to “yes a lot”, as described by others [25,36]. Participants marked along the line indicating their feelings, which were then assigned a quantifiable value by measuring from the beginning of the line to where the participant had marked on the 10 cm line [25]. Each participant was asked to fill out the VAS questionnaire in the morning after overnight fasting conditions and before any food was taken, for 5 days at baseline and during the last week of the intervention. With the 5-day results, before and after the intervention, a respective average was built for the analysis.

### 2.17. Statistical Analysis

For the qualitative variables, relative and absolute frequencies were calculated for each of the intervention groups and Pearson’s chi-square and Fisher’s exact test were applied for frequencies less than 5, to compare the groups. For the analysis of diet and subjective and objective satiety variables, summary measures were calculated, and an ANOVA of repeated measures was applied in the Rstudio interface, in addition to the VAS and ghrelin levels in each of the intervention groups. A Cumming estimation plot was constructed with the baseline and end data of the subjects using the ESTIMATION STATS program, according to the guidelines of Joses Ho et al. [37] to calculate the effect size.

### 2.18. Ethical Aspects

The clinical trial obtained the approval of the bioethics committee of the University Research Headquarters of the University of Antioquia. Additionally, the ethical principles complied with the Declaration of Helsinki and the CIOMS guidelines. All the participants knew and previously accepted the protocol and signed the informed consent.

## 3. Results

### 3.1. Characteristics of the Intervened Population and Diet Analysis

Of the 144 screened people, 109 were enrolled and randomized. During the screening period, it was found that 32% did not meet the inclusion or had some exclusion criteria. During the follow-up process, four dropouts (3.8%) occurred, with 105 participants completing the study, 35 in each group. The participation of women was higher than men (65.8% and 60%, respectively), and the middle socioeconomic stratum was more frequent. The mean age was 28 years, and the most prevalent level of education was professional.

In relation to nutrient intake, no significant differences (*p* > 0.05) were found between the intervention groups, neither in time nor in the interaction time treatment (Table 1). While the intake of protein was kept constant (*p* > 0.05) between the groups, participants consumed significantly (*p* < 0.05) less kilocalories, total fat, saturated, monounsaturated, polyunsaturated fatty acids, carbohydrates, and fiber over time. As expected, dietary cholesterol increased in the egg groups, compared to egg-whites (*p* = 0.00).

### 3.2. Effects on Anthropometric Variables, Physical Activity, and Blood Pressure

No differences were observed in relation to the intervention time for any of the anthropometric variables. Significant differences (*p* < 0.05) were observed between treatment groups for waist circumference, height, and weight. But no significant changes (*p* > 0.05) were found when time x treatment was evaluated, neither for BMI nor for physical activity or blood pressure, see Table 2. When classified according to BMI, no significant differences were found between obesity and normal weight (*p* > 0.05). Effects on blood lipids and glucose were not different between the groups (*p* > 0.05; for more information see [34]).

### 3.3. Effects on Subjective and Objective Satiety

No significant differences (*p* > 0.05) were observed in the VAS of the subjective measures of satiety and in the objective measure of it (ghrelin), nor in treatment time and treatment time interaction for the analysis using repeated measures ANOVA, see Table 3.

**Figure 1:**
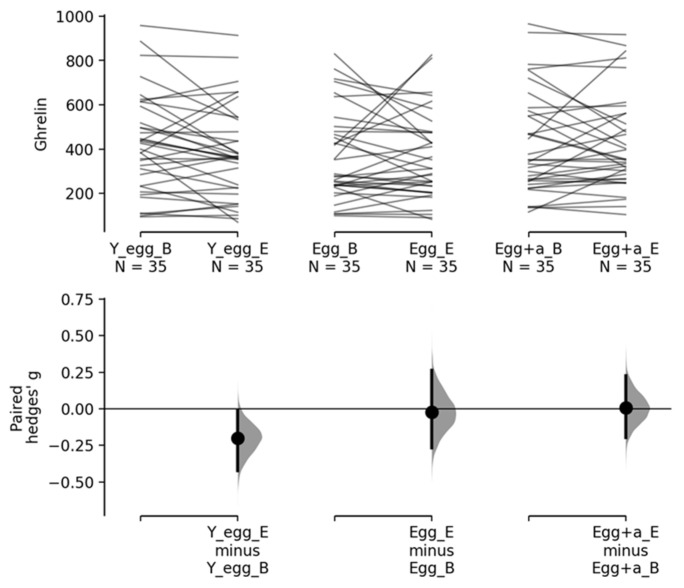
Ghrelin levels as a measurement of objective satiety in the intervention groups. Y_egg_B: Egg whites Baseline. Y_egg_E: Egg whites End-8 weeks. Egg_B: egg Baseline. Egg_E: Egg End-8 weeks. Egg + a_B: Egg + annatto baseline. Egg + a_E: Egg + annatto end-8 weeks. The paired Hedges’ g for three comparisons are shown in the above Cumming estimation plot. The raw data is plotted on the upper axes; each paired set of observations is connected by a line. On the lower axes, each paired mean difference is plotted as a bootstrap sampling distribution. Mean differences are depicted as dots; 95% confidence intervals are indicated by the ends of the vertical error bars.

**Figure 2:**
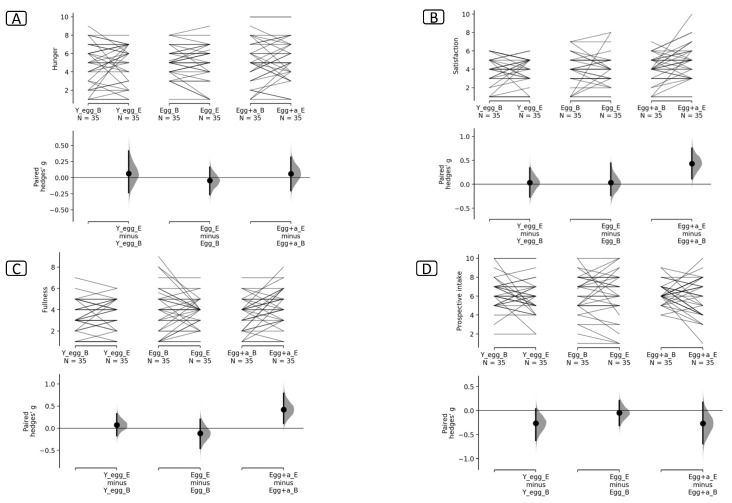
Visual analog scale of (**A**) hunger, (**B**) satisfaction, (**C**) fullness, and (**D**) prospective food intake. Y_egg_B: Egg whites Baseline. Y_egg_E: Egg whites End-8 weeks. Egg_B: egg Baseline. Egg_E: Egg End-8 weeks. Egg + a_B: Egg + annatto baseline. Egg + a_E: Egg +annatto End-8 weeks. The paired Hedges’ g for three comparisons are shown in the above Cumming estimation plot. The raw data is plotted on the upper axes; each paired set of observations is connected by a line. On the lower axes, each paired mean difference is plotted as a bootstrap sampling distribution. Mean differences are depicted as dots; 95% confidence intervals are indicated by the ends of the vertical error bars.

As indicated, the effect size was calculated using Hedges’ G, which shows that no changes were observed over time for any of the treatment groups in the objective satiety variable (Ghrelin), see Figure 1, but for the subjective measurements on the VAS, significant changes were found in satisfaction (0.431 [0.113, 0.753]. *p* = 0.009) and fullness 0.384 [0.11, 0.721] *p* = 0.009) for the egg + annatto group after the intervention (see Figure 2).

## 4. Discussion

According to what was observed, we found a homogeneous population through the intervention groups without significant changes in anthropometric variables, evidence of the correct random assignment and of the balance between the intervention groups that also applies to what is observed in Table 2 on BMI. In the same way, no differences were found in blood pressure, which agrees with other studies evaluating egg consumption effects [38,39]. As expected, there were no differences in physical activity, a control variable used to ensure participants would maintain their lifestyle through the study.

Similarly, participants were asked to maintain their habitual diet, except for the consumption of additional eggs or egg-whites, or annatto. The diet analysis shows that there were no differences in relation to protein intake neither in time, nor in treatment and in the interaction of time and treatment. However, fat and carbohydrate intake decreased over time in all groups. As described before, protein has more satiating effects than fat, and fat more than carbohydrates [40,41]. The lipid content present in the egg yolk was expected to provide more satiating effects, compared to egg-whites; however, given that protein intake was constant over time, but total fat intake decreased in all groups, it is more consistent that protein had driven similar satiety effects between the groups. In addition, the contribution of protein from egg-whites (control group) may also have been important, diminishing the possibility of finding differences in satiety.

Differences in macronutrient intake were only observed over time, but were similar in all groups, this evidences that the ad libitum intake of these nutrients was balanced for the groups and is consistent with the similar total kilocalories registered, which also decreased significantly over time (*p* = 0.006), but without differences by treatment. This agrees with the study by Missimer et al., who also reported the same contribution of kilocalories for their intervention groups [25]. However, they reported greater satiating effect prior to eating dinner, when participants consumed eggs for breakfast, compared to an oatmeal breakfast. Unlike our study, these authors observed increases in the percentage of calories from protein and fat after the egg period. Although the study by Missimmer et al. did not control the diet of the participants, similar to our study, there are some important differences in the study design that could have contributed to the differences in the observed results of satiety after consuming eggs: first, the crossover design versus the parallel design used in our study, and perhaps it was more important to have evaluated satiety at several points before and after the egg breakfast, reporting differences after egg consumption. In this study, satiety was evaluated only before breakfast.

In relation to the changes in the different types of fatty acids (saturated-SFA, monounsaturated-MUFA and polyunsaturated-PUFA), significant changes were evident over time and in treatment (see Table 1). We observed that participants consuming eggs or eggs + annatto tended to have, on average, less reductions in SFA, MUFA and PUFA, than people consuming egg-whites. Other interventions with eggs have reported increases in SFA and MUFA, with no differences on PUFA [25]. And as expected, there were significant increases (*p* < 0.000) in dietary cholesterol in the groups consuming whole eggs, given the percentage of cholesterol provided by the yolk. These contributions to the diet are important, especially cholesterol and PUFA for biological processes [42,43]. In this regard, although they are not the components of the diet that provide the greatest satiety such as proteins, they are considered important with respect to changes in satiety hormones and in the perception of hunger [44,45,46].

No significant differences (*p* > 0.05) were observed in the VAS of the subjective measures of satiety and in the objective measure of satiety (ghrelin), nor in time, treatment and treatment time interaction for the analysis using repeated measures ANOVA, this differs from what was found in other studies [9,25]. In the case of ghrelin, this can be explained if one takes into consideration that this hormone has been more associated with changes in weight gain or carbohydrate reduction in diet [47,48]; although others have shown that ghrelin is strongly suppressed after protein intake compared to carbohydrates [49,50] as the type of intervention carried out in the aforementioned studies [9,25]. We already discussed that protein intake in our study was the same across the treatment groups, therefore, it is expected that ghrelin levels were also similar. In addition, the time points where ghrelin was measured and the VAS for the assessment of satiety was applied differ between those studies and our study. Those changes in satiety were observed acutely as previously mentioned [25].

Given the previous results, the size of the effect was calculated using Hedges’ G, which shows that no changes were observed in the times for any of the treatments in ghrelin (Figure 1), but for the subjective measurement we found significant changes in satisfaction (0.431 [0.113, 0.753], *p* = 0.009) and fullness 0.384 [0.11, 0.721] *p* = 0.009) for the egg + annatto group (Figure 2). It is important to mention that although we could not find significant differences in the averages for these variables in the repeated measures ANOVA, we found that the effect size of the differences was clinically significant. It has been demonstrated that changes in satiety perception could be due to various factors such as energy density, macronutrient composition, physical structure, and sensory quality of the food ingested [51]. Some studies have suggested the role of appetite-suppressing mechanisms unrelated to the antioxidant activity of the foods consumed [52,53]. Although the egg + annatto group could have consumed more antioxidants, the clinically significant satiating effects found in this group may be related to the bioavailability of other components, such as fiber, present in the annatto [54]. This finding could represent an important alternative in the searching to improve satiety perception in individuals through other components or activities present in antioxidant-rich foods, and it may also show that eggs can be a good matrix for fat-soluble antioxidants, such as annatto, to improve their biological effects.

Finally, it is also important to consider that other cultural and lifestyle habits may affect the relation between satiety and energy intake and expenditure, that further will affect body weight. The current obesity epidemic suggests that this relationship is disrupted [55]. Therefore, one should not expect that a person feeling a little more satiated would necessarily decrease his/her energy intake and decrease body weight in a few weeks, this relationship seems to be more complex.

## 5. Conclusions

The results showed that consumption of two eggs a day for 8 weeks did not modify body weight, blood pressure, or satiety when compared to egg-white consumption. It is possible that the similar protein intake observed for all three groups may have not rendered the possibility of finding significant differences. However, other components present in antioxidants like annatto could have a role in the size of the effect observed in the satisfaction and fullness after consuming eggs enriched with annatto.

This is important because it highlights the biological effect of antioxidant-rich foods or their complementary components in lipid matrices such as eggs on satiety, promoting further studies evaluating antioxidant consumption effects on satiety and alternatives to improve their bioavailability.

It is also important to highlight the beneficial effects of egg consumption where, besides providing a lipid matrix to improve the bioavailability of lipophilic antioxidants, the egg itself contain antioxidants and other nutrients that also may have a biological effect on satiety that need to be explored at the molecular level.

## Figures and Tables

**Table 1:** Diet analysis in the population according to treatment groups.

	Egg Whites		Egg		Egg + Annatto				
	Median (±SD)		Median (±SD)		Median (±SD)		p^1^	p^2^	p^3^
	Baseline	8 Weeks	Baseline	8 Weeks	Baseline	8 Weeks			
Protein (g)	69.3 (13.4)	64.8 (13.4)	71.4 (15.9)	69.1 (11.9)	71.5 (16.7)	70.7 (11.9)	0.192	0.193	0.741
Total fat (g)	64.6 (15)	53.6 (15.3)	69.2 (18.1)	64.8 (13.9)	65 (15.6)	63.5 (13)	0.008	0.008	0.07
Saturated fatty acids (g)	22.9 (6.39)	18.8 (5.84)	24.8 (7.11)	23.0 (6.47)	22.9 (6.42)	22 (5.33)	0.008	0.014	0.219
Monounsaturated fatty acids (g)	25.8 (6.30)	21.8 (7.26)	27.2 (7.44)	25.4 (5.19)	26.1 (6.54)	25.3 (5.22)	0.012	0.026	0.14
Polyunsaturated fatty acids (g)	11.1 (2.67)	9.69 (2.73)	12.2 (4.14)	11.3 (2.73)	11.5 (2.82)	11.2 (2.68)	0.037	0.033	0.37
Carbohydrates (g)	236 (69.8)	211 (52.9)	249 (80)	219 (66.3)	247 (70.9)	225 (56.3)	0.008	0.599	0.911
Cholesterol (mg)	497 (209)	152 (76.5)	482 (220)	559 (43.1)	465 (207.1)	549 (64.2)	0.000	0.000	0.000
Fiber (g)	14.8 (5.06)	14.2 (4.85)	15.4 (4.81)	14.1 (3.73)	15 (4.67)	13.0 (4.92)	0.03	0.622	0.677
Energy (kcal)	1802 (389)	1589 (365)	1890 (494)	1718 (377)	1841 (432)	1748 (350)	0.006	0.242	0.568

Repeated-measures ANOVA. p^1^: time, p^2^: treatment, p^3^: time × treatment.

**Table 2:** Anthropometry, physical activity, and blood pressure in the studied population.

	Egg Whites		Egg		Egg + Annatto				
	Median (±SD)		Median (±SD)		Median (±SD)		p^1^	p^2^	p^3^
	Baseline	8 Weeks	Baseline	8 Weeks	Baseline	8 Weeks			
Waist circumference (cm)	79.8 (8.39)	80.5 (8.39)	83.7 (9.24)	83.7 (8.67)	82.5 (6.08)	83.7 (8.67)	0.89	0.03	0.954
Body weight (Kg)	61.8 (9.1)	61.6 (8.3)	66.6 (10.1)	66.3 (10.7)	63.3 (8.8)	63.7 (8.4)	0.971	0.009	0.973
Height (m)	1.62 (0.08)	1.62 (0.08)	1.65 (0.08)	1.65 (0.08)	1.62 (0.1)	1.62 (0.1)	0.971	0.009	0.973
BMI (kg/m^2^)	23.5 (2.80)	23.5 (2.69)	24.4 (2.81)	24.3 (2.96)	24 (2.77)	24.2 (2.79)	0.998	0.202	0.953
Physical activity (Mets)	1124 (802)	842 (653)	1124 (1049)	1322 (1099)	1133 (798)	1027 (807)	0.11	0.272	0.84
Systolic blood pressure (mm Hg)	110 (11.9)	110 (12.7)	110 (9.8)	108 (9.4)	108 (7.9)	106 (9.4)	0.43	0.188	0.863
Diastolic blood pressure (mm Hg)	64.9 (7.7)	64.6 (6.9)	66.6 (6.8)	64.5 (5.4)	66.1 (7.7)	64.3 (7.8)	0.143	0.797	0.712

Repeated-measures ANOVA. p^1^: time, p^2^: treatment, p^3^: time × treatment.

**Table 3:** Ghrelin levels and satiety scores on the visual analogue scale (VAS) by intervention group.

	Egg Whites		Egg		Egg + Annatto		p^1^	P^2^	P^3^
	Median (±SD)		Median (±SD)		Median (±SD)				
	Baseline	8 Weeks	Baseline	8 Weeks	Baseline	8 Weeks			
Ghrelin (pg/mL)	425 (221)	397 (227)	363 (202)	359 (194)	422 (231)	424 (214)	0.75	0.581	0.562
Hunger	4.77 (2.35)	4.91 (2.13)	5.23 (1.83)	5.01 (2.02)	5.2 (2.47)	5.34 (2.27)	0.945	0.503	0.85
Satisfaction	3.8 (2.01)	3.86 (1.88)	4.2 (2)	4.57 (2.28)	4.17 (1.79)	4.8 (1.94)	0.201	0.098	0.728
Fullness	3.8 (2.18)	3.69 (1.86)	3.99 (2.17)	4.57 (2.5)	3.74 (1.84)	4.49 (1.99)	0.151	0.347	0.494
Prospective intake	6.26 (2)	5.77 (2.02)	6.17 (2.13)	6.06 (2.51)	6.17 (1.77)	5.86 (2.05)	0.123	0.201	0.679

Repeated-measures ANOVA. p^1^: time, p^2^: treatment, p^3^: time × treatment.

## Data Availability

The data presented in this study are available on request from the corresponding author. The data are not publicly available as analyses are still underway for additional publications.
